# Supplementary material for: Cellular mechanical properties in response to environmental viscosity imaged by Brillouin Microscopy
Source: Commun Biol. 2025 Nov 24;8:1645. doi: 10.1038/s42003-025-09032-5 (PMC12644817; doi:10.1038/s42003-025-09032-5)
Supplement: Supplementary file 2 — Supplementary Materials [file 42003_2025_9032_MOESM2_ESM.pdf]

Supplementary Materials for

Cellular mechanical properties in response to environmental viscosity imaged by Brillouin Microscopy

Chenchen Handler[1,4], Giulia Zanini[2,3], Ian M. Smith[2], Kimberly M. Stroka[2], Giuliano Scarcelli[2] and Claudia Testi*[2,5]

1: Department of Mechanical Engineering, A. James Clark School of Engineering, University of Maryland, College Park, MD 20742, USA

2: Fischell Department of Bioengineering, A. James Clark School of Engineering, University of Maryland, College Park, MD 20742, USA

3: *Current address:* CrestOptics S.p.A., Via Di Torre Rossa, 66, 00165, Roma, Italy

4: Laboratory of Cell Biology, Center for Cancer Research, National Cancer Institute, National Institutes of Health, Bethesda, Maryland

5: Center for Life Nano- and Neuro- Science, Istituto Italiano di Tecnologia, Viale Regina Elena 291, Rome 00161, Italy

Correspondance: claudia.testi@iit.it

Supplementary Tables

	ν_B @ 660 nm [GHz]	Γ_B @ 660 nm [GHz]
**Distilled Water**	5.932 ± 0.004	0.42 ± 0.01
**Normal cell medium**	6.013 ± 0.003	0.43 ± 0.01
**LV medium**	6.019 ± 0.003	0.42 ± 0.01
**HV medium**	6.120 ± 0.004	0.53 ± 0.02
**N2**	5.75 ± 0.01	0.79 ± 0.04
**S3**	6.28 ± 0.01	1.518 ± 0.07
**Methanol**	4.590 ± 0.003	0.236 ± 0.008

Supplementary Table 1: List of FWHMs obtained from SBM[32] that have been used as theoretical values for Γ_B of Figure 2. Values are given as mean ± SD. Each of them has been obtained by averaging N=3 independent measurements (each of which contained 100 identical measurements). All materials have been measured at room temperature, with a 40x objective (NA = 0.95), at a wavelength of 780 nm; we converted the results at 660 nm ($\nu_B^{660\,nm} = \nu_B^{780\,nm} * \frac{660}{780}$; $\Gamma_B^{660\,nm} = \Gamma_B^{780\,nm} * \left(\frac{660}{780}\right)^2$).

	n	ρ (g/mL)	η_{Shear} (cP)
**Distilled Water**	1.333	1	1.06
**Normal cell medium**	1.3362	1.016	1.06
**LV medium**	1.3362	1.016	1.06
**LV medium (37°C)**	1.3362	1.016	0.85
**HV medium**	1.3451	1.016	12.35
**HV medium (37°C)**	1.3451	1.016	9.90
**N2**	1.4285	0.758	2.0
**S3**	1.4752	0.864	3.3
**Methanol**	1.326	0.792	0.54

Supplementary Table 2: List of experimentally obtained materials properties, used to calculate η_{bulk} in Equation 4 and Figure 3B.

Supplementary Figures

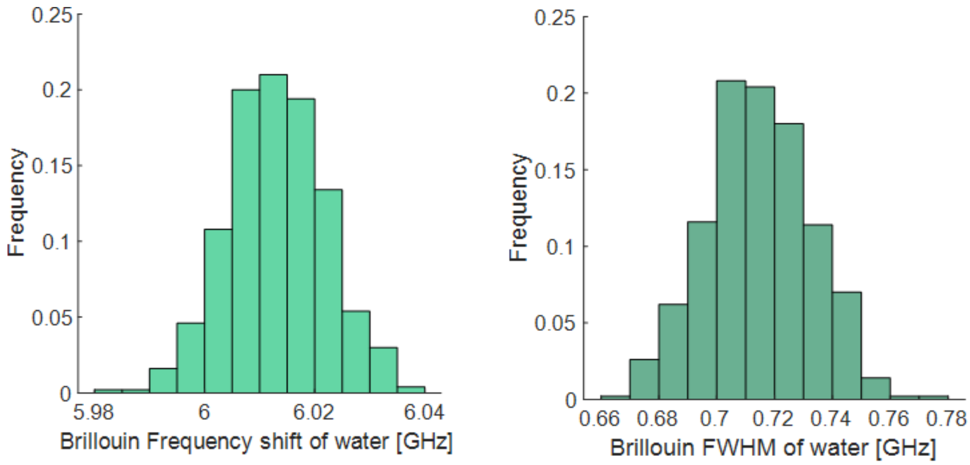

Supplementary Figure 1: Precision of Brillouin shift and FWHM of our custom-made Brillouin Microscope. The precision of our Brillouin Microscope was 9 MHz for the Brillouin shift and 18 MHz for the Brilllouin FWHM, measured with the SD of the water Brillouin spectra (N=500 spectra, 100 ms exposure time, 50 mW power on sample plane).

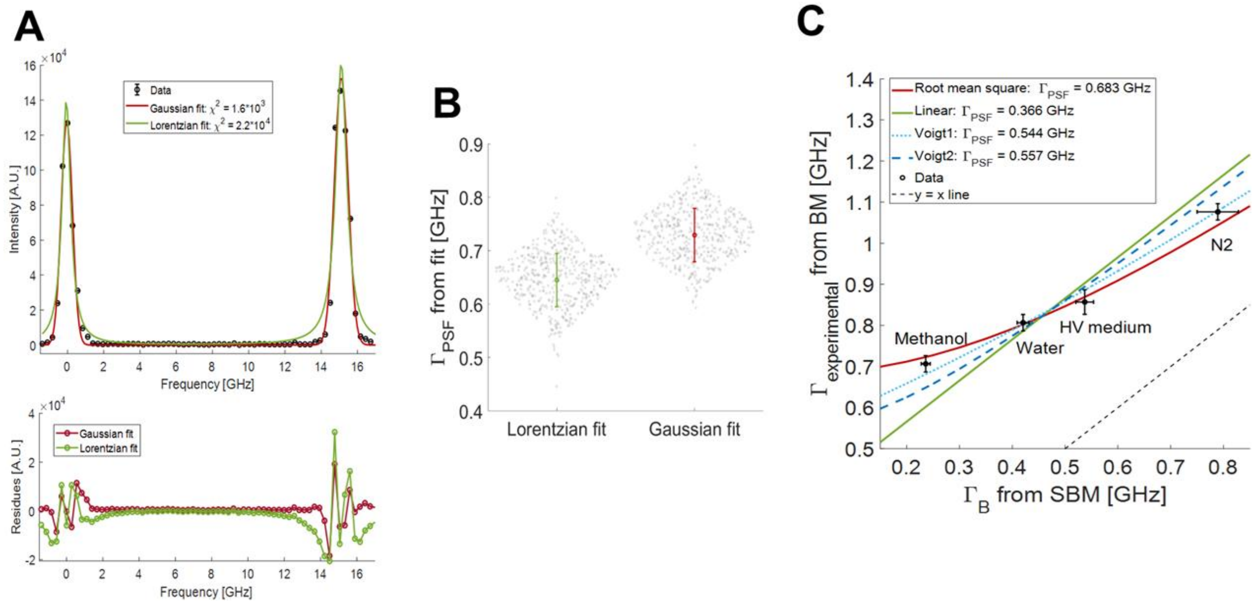

Supplementary Figure 2: Rayleigh peaks FWHMs matched the value found from calibration materials. Panel A: Elastic Rayleigh peaks were imaged on the camera by lowering the laser intensity to the minimum and opening the slits of the spectrometer; we fitted the resulting spectra with either a Gaussian (red line) or Lorentzian (green line) to find out which one fitted the best. Gaussian function was more accurate in reproducing the instrumental line shape, especially at the tails of the signal; χ^2 was lower and the residues (lower panel) were closer to 0 with respect to Lorentzian, confirming that the VIPAs function response cannot be described as a pure Lorentzian. **Panel B:** Despite the differences, both fits resulted in similar full widths at half maximum. Data here are shown as mean ± SD performed on 500 spectra. **Panel C:** The FWHMs from the elastics fit matched the value found from Gaussian convolution (red solid line) of 683 MHz. All the other models (linear and Voigt profiles) resulted in a sever underestimation of the instrumental resolution.

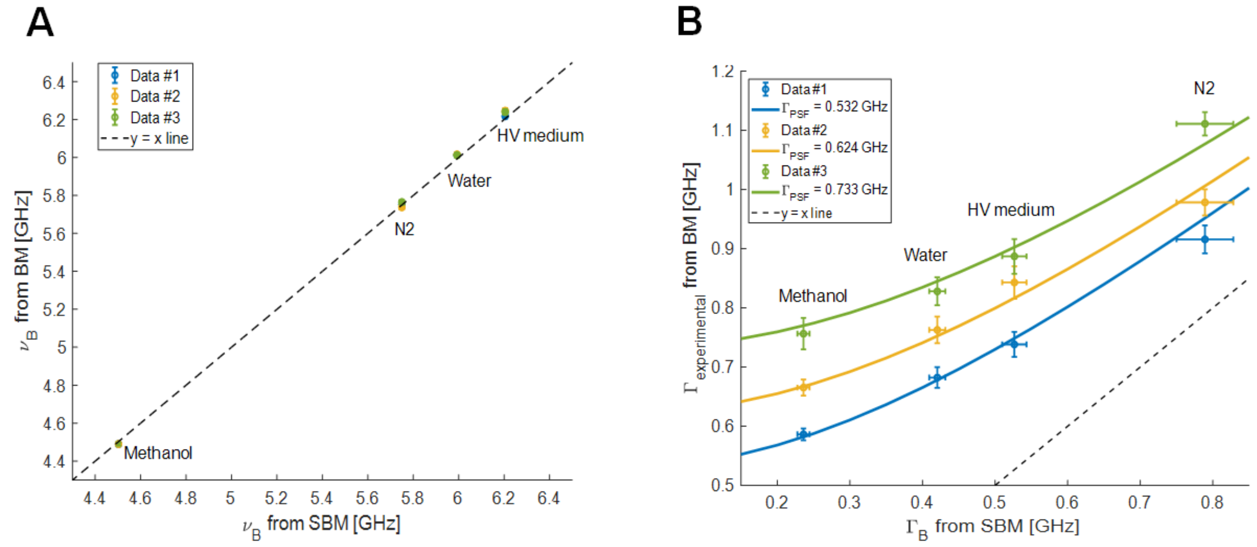

Supplementary Figure 3: comparison between BM data taken at different days (blue, yellow and green datapoints). Panel A: Brillouin shift values of calibration materials imaged with the BM were very reproducible between different experimental days. Moreover, their values always matched the ones of the SBM, confirming that the convolution operation (equation 2) affects only the widths. **Panel B:** On the other hand, Brillouin FWHMs of calibration materials obtained with BM were very different from day to day and were calibration sensitive. Their values were always much higher than the ones of SBM and followed a Gaussian convolution profile (continuous line) that yielded Γ_{PSF} ranging from 530 to 730 MHz, different from day to day. Black line: y=x line. Data points are shown as mean ± SD, performed over n=500 repeated measurements for 3 different days.

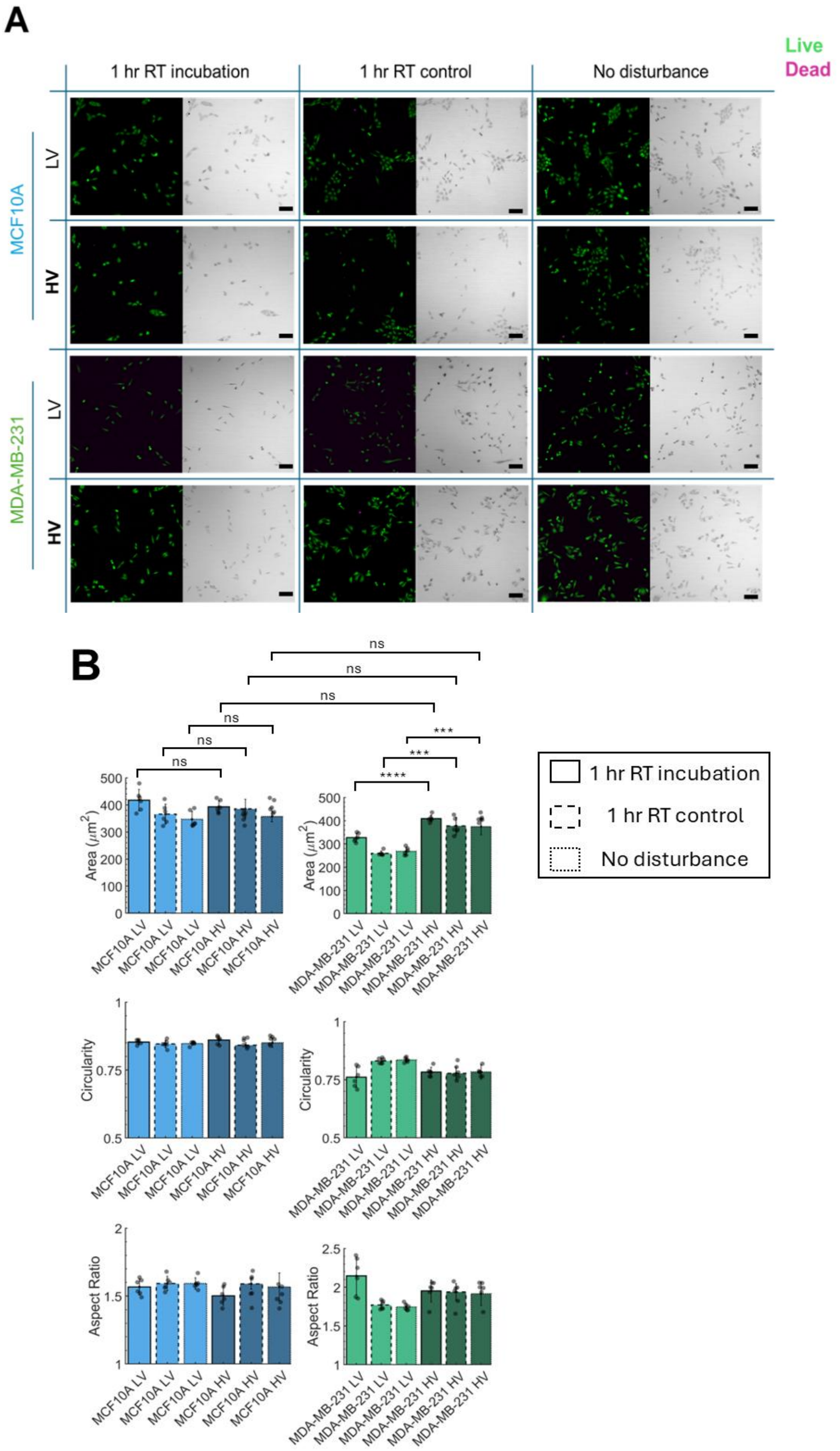
A
Live
Dead
1 hr RT incubation
1 hr RT control
No disturbance
MCF10A
MDA-MB-231
LV
HV
B
1 hr RT incubation
1 hr RT control
No disturbance
ns
****
***
Area (μm²)
Circularity
Aspect Ratio
MCF10A LV
MCF10A HV
MDA-MB-231 LV
MDA-MB-231 HV

Supplementary Figure 4: live/dead microscopy images of cells in LV and HV media. Panel A: fluorescence and brightfield images of MCF10A and MDA-MB-231 cells under LV and HV media after 1 hr RT incubation, 1 hr RT control, and no disturbance. Live cells are labeled in green and dead cells are labeled in magenta. Scale bars = 100 microns. **Panel B:** Average cellular area, circularity, and aspect ratio quantified under LV and HV media after after 1 hr RT incubation (solid outline), 1 hr RT control (dashed outline), and no disturbance (dotted outline). Statistical significance performed using unpaired t-test with Welch's correction, not assuming equal SDs. ***: $p<0.001$; ****: $p<0.0001$; ns = not significant. Data here are shown as mean ± SD, derived from N = 6 independent experiments.
