# Supplementary material for: Cellular mechanical properties in response to environmental viscosity imaged by Brillouin Microscopy
Source: Commun Biol. 2025 Nov 24;8:1645. doi: 10.1038/s42003-025-09032-5 (PMC12644817; doi:10.1038/s42003-025-09032-5)
Supplement: Supplementary file 5 — Reporting Summary [file 42003_2025_9032_MOESM5_ESM.pdf]

nature portfolio

Corresponding author(s): Testi Claudia

Last updated by author(s): Sep 29, 2025

Reporting Summary

Nature Portfolio wishes to improve the reproducibility of the work that we publish. This form provides structure for consistency and transparency in reporting. For further information on Nature Portfolio policies, see our Editorial Policies and the Editorial Policy Checklist.

Statistics

For all statistical analyses, confirm that the following items are present in the figure legend, table legend, main text, or Methods section.

n/a	Confirmed	
☐	☒	The exact sample size (*n*) for each experimental group/condition, given as a discrete number and unit of measurement
☐	☒	A statement on whether measurements were taken from distinct samples or whether the same sample was measured repeatedly
☐	☒	The statistical test(s) used AND whether they are one- or two-sided<br>*Only common tests should be described solely by name; describe more complex techniques in the Methods section.*
☒	☐	A description of all covariates tested
☐	☒	A description of any assumptions or corrections, such as tests of normality and adjustment for multiple comparisons
☐	☒	A full description of the statistical parameters including central tendency (e.g. means) or other basic estimates (e.g. regression coefficient) AND variation (e.g. standard deviation) or associated estimates of uncertainty (e.g. confidence intervals)
☐	☒	For null hypothesis testing, the test statistic (e.g. *F*, *t*, *r*) with confidence intervals, effect sizes, degrees of freedom and *P* value noted<br>*Give P values as exact values whenever suitable.*
☒	☐	For Bayesian analysis, information on the choice of priors and Markov chain Monte Carlo settings
☒	☐	For hierarchical and complex designs, identification of the appropriate level for tests and full reporting of outcomes
☒	☐	Estimates of effect sizes (e.g. Cohen's *d*, Pearson's *r*), indicating how they were calculated

Our web collection on statistics for biologists contains articles on many of the points above.

Software and code

Policy information about availability of computer code

Data collection	A home built LabView (National Instruments, ver. 2021) acquisition program was used to acquire both brightfield images and the Brillouin spectra.
Data analysis	Brillouin spectra have been analuzed with custom-made MATLAB codes. Statistical analysis was performed in GraphPad Prism 8 (see Methods)

For manuscripts utilizing custom algorithms or software that are central to the research but not yet described in published literature, software must be made available to editors and reviewers. We strongly encourage code deposition in a community repository (e.g. GitHub). See the Nature Portfolio guidelines for submitting code & software for further information.

Data

Policy information about availability of data

All manuscripts must include a data availability statement. This statement should provide the following information, where applicable:

- Accession codes, unique identifiers, or web links for publicly available datasets
- A description of any restrictions on data availability
- For clinical datasets or third party data, please ensure that the statement adheres to our policy

Source data for graphs and charts that support the findings of this study are available in Numerical source data (Supplementary Data 5).

Research involving human participants, their data, or biological material

Policy information about studies with human participants or human data. See also policy information about sex, gender (identity/presentation), and sexual orientation and race, ethnicity and racism.

Reporting on sex and gender	N/A
Reporting on race, ethnicity, or other socially relevant groupings	N/A
Population characteristics	N/A
Recruitment	N/A
Ethics oversight	N/A

Note that full information on the approval of the study protocol must also be provided in the manuscript.

Field-specific reporting

Please select the one below that is the best fit for your research. If you are not sure, read the appropriate sections before making your selection.

☒ Life sciences ☐ Behavioural & social sciences ☐ Ecological, evolutionary & environmental sciences

For a reference copy of the document with all sections, see nature.com/documents/nr-reporting-summary-flat.pdf

Life sciences study design

All studies must disclose on these points even when the disclosure is negative.

Sample size	Sample size and experimental replication scheme were chosen based on our previous experience, and to be reasonably large, from at least n=3 independent biological replicates.
Data exclusions	No data were excluded from the analysis.
Replication	All replications were successful and gave similar results. All microscopy experiments were repeated independently at least three times (from a fresh culture flask of cells, with fresh aliquotes of ingredients).
Randomization	All cells were selected at random, in random fields of view during data acquisition. Identical and independent culture wells were prepared for each experimental condition, therefore no special allocation procedure was necessary.
Blinding	Investigators were not blined during data acquisition and analysis.

Reporting for specific materials, systems and methods

We require information from authors about some types of materials, experimental systems and methods used in many studies. Here, indicate whether each material, system or method listed is relevant to your study. If you are not sure if a list item applies to your research, read the appropriate section before selecting a response.

Materials & experimental systems

n/a	Involved in the study	
☒	☐	Antibodies
☐	☒	Eukaryotic cell lines
☒	☐	Palaeontology and archaeology
☒	☐	Animals and other organisms
☒	☐	Clinical data
☒	☐	Dual use research of concern
☒	☐	Plants

Methods

n/a	Involved in the study	
☒	☐	ChIP-seq
☒	☐	Flow cytometry
☒	☐	MRI-based neuroimaging

Eukaryotic cell lines

Policy information about cell lines and Sex and Gender in Research

Cell line source(s)	MCF10A cells were a gift from Michele Vitolo from the University of Maryland, School of Medicine. MDA-MB-231 cells were purchased from ATCC (HTB-26).
Authentication	Cells were not authenticated. Only cells with low passage number (<10) were used after obtaining them directly from the supplier.
Mycoplasma contamination	Cells lines have been tested for Mycoplasma contamination with the MycoFluor Mycoplasma Detection Kit (Thermofisher) and were confirmed to be negative for mycoplasma.
Commonly misidentified lines (See ICLAC register)	None

Plants

Seed stocks	N/A
Novel plant genotypes	N/A
Authentication	N/A
